# Machine learning algorithms predict canine structural epilepsy with high accuracy

**DOI:** 10.3389/fvets.2024.1406107

**Published:** 2024-07-22

**Authors:** Thomas Flegel, Anja Neumann, Anna-Lena Holst, Olivia Kretzschmann, Shenja Loderstedt, Carina Tästensen, Sarah Gutmann, Josephine Dietzel, Lisa Franziska Becker, Theresa Kalliwoda, Vivian Weiß, Madlene Kowarik, Irene Christine Böttcher, Christian Martin

**Affiliations:** ^1^Department for Small Animals, Veterinary Faculty, Leipzig University, Leipzig, Germany; ^2^Center for Scalable Data Analytics and Artificial Intelligence (ScaDS.AI), Leipzig University, Leipzig, Germany

**Keywords:** dog, seizures, artificial intelligence, Random Forest, Bayesian Network, feature selection

## Abstract

**Introduction:**

Clinical reasoning in veterinary medicine is often based on clinicians’ personal experience in combination with information derived from publications describing cohorts of patients. Studies on the use of scientific methods for patient individual decision making are largely lacking. This applies to the prediction of the individual underlying pathology in seizuring dogs as well. The aim of this study was to apply machine learning to the prediction of the risk of structural epilepsy in dogs with seizures.

**Materials and methods:**

Dogs with a history of seizures were retrospectively as well as prospectively included. Data about clinical history, neurological examination, diagnostic tests performed as well as the final diagnosis were collected. For data analysis, the Bayesian Network and Random Forest algorithms were used. A total of 33 features for Random Forest and 17 for Bayesian Network were available for analysis. The following four feature selection methods were applied to select features for further analysis: Permutation Importance, Forward Selection, Random Selection and Expert Opinion. The two algorithms Bayesian Network and Random Forest were trained to predict structural epilepsy using the selected features.

**Results:**

A total of 328 dogs of 119 different breeds were identified retrospectively between January 2017 and June 2021, of which 33.2% were diagnosed with structural epilepsy. An overall of 89,848 models were trained. The Bayesian Network in combination with the Random feature selection performed best. It was able to predict structural epilepsy with an accuracy of 0.969 (sensitivity: 0.857, specificity: 1.000) among all dogs with seizures using the following features: age at first seizure, cluster seizures, seizure in last 24 h, seizure in last 6 month, and seizure in last year.

**Conclusion:**

Machine learning algorithms such as Bayesian Networks and Random Forests identify dogs with structural epilepsy with a high sensitivity and specificity. This information could provide some guidance to clinicians and pet owners in their clinical decision-making process.

## 1 Introduction

Medical decisions on diagnostic interventions, interpretation of results of diagnostic tests and treatment protocols in an individual case are currently heavily influenced by the attending clinician’s level of veterinary training, participation in continuing education courses, level of knowledge of pertinent scientific literature and personal experiences gained from prior cases. That may result in completely different decisions in cases that are presented with identical clinical signs.

There are first attempts to add more objective information to the medical decision-making process using artificial intelligence (AI) (1–8). Several of those are centered around forecasting seizures. A support vector machine algorithm was investigated to predict seizures in 5 dogs based on intracranial electroencephalography (EEG) tracings (4). Authors highlight the importance of correct feature selection. Another study describes the training of a subject-specific deep-learning convolutional neural network model to predict seizures in 4 dogs based on ambulatory intracranial EEG recordings (8). This model forecasted seizures with a mean sensitivity of 0.79. Similarly, a precision-recall genetic algorithm in line with a probabilistic support vector machine classifier was used for seizure forecasting in canine epilepsy, again based on intracranial EEG recordings in 3 dogs (2).

Another focus is the prediction of epilepsy types with the regard to the underlying etiology. Epilepsy can be classified as idiopathic or structural. The latter is defined as epileptic seizures that are caused by a structural intracranial pathology, whereas the underlying cause is either genetic or not known yet in idiopathic epilepsy (9). Abani et al. (1) investigated the possibility to use ChatGPT to determine the underlying pathology causing seizures in dogs based on age, clinical signs, seizure characteristics and results of the neurological examination (2023). ChatGPT correctly identified dogs with idiopathic epilepsy based on the clinical history in 2 out of 5 cases. Adding results of the clinical examination improved the correct rate to 4 out of 5 dogs. Cases with structural brain abnormalities were correctly diagnosed in 1 out of 5 cases just based on clinical history, which could again be improved to 2 out of 5 cases by adding the results of the clinical examination. Cases with paroxysmal dyskinesia, however, were not identified by ChatGPT.

In this study we evaluated two machine learning algorithms to detect structural epilepsy in dogs.

## 2 Materials and methods

### 2.1 Data acquisition

This study is based on data that has been collected retrospectively and prospectively. In the first phase, the hospital database of the Department for Small Animals at Leipzig University was screened for dogs using the following search terms: astrocytoma, adenoma, adenocarcinoma, encephalitis, epilepsy, esthesioneuroblastoma, glioma, hydrocephalus, intoxication, lymphoma, meningioma, neoplasia, neuroblastoma, postictal, seizures, shunt, status, and cluster. The following information was extracted for dogs being identified: age, sex, breed, body weight, age at first seizure, first symptoms observed by the owner, type of seizure, number of seizures before first presentation, observed cluster of seizures, observed status epilepticus, results of neurological examination, results of diagnostic investigation, survival time, and final diagnosis. In a second phase, the features identified to be most relevant in the first phase based on the validation of different feature selection methods were collected prospectively for patients being presented to the hospital from this time point on.

### 2.2 Data preprocessing

Dogs were included retrospectively as well as prospectively. Data sets of both groups of dogs were not identical. In order to integrate the data from both groups of dogs, the following parameters were chosen for further analysis: age at presentation, body weight, sex including castration status, age at first seizure, duration of seizure history, seizure in last 24 h, seizures in last week (excluding those in the last 24 h), seizures in last month (excluding those in the last week), seizures in last 6 months (excluding those in the last month), seizures in last year (excluding those in the last 6 months), neurological deficits at presentation, history of cluster seizures, history of status epilepticus, lateralized neurological deficit at presentation, first clinical signs observed by the owner, and seizure type (generalized tonic–clonic, partial, both). Entries with missing values were removed. Table keys were adapted and unified, the values were cleaned. First clinical signs were grouped to paroxysmal events, abnormal behavior, vocalization, abnormal coordination, abnormal motor movements, gastrointestinal signs, recumbency, salivation and head tremor. All classes with less than 5 members were labeled with other clinical signs. The resulting table was extended by three further columns “age in days”, “structural brain disease” (yes or no) “and “weight groups” to represent breeds. The final resulting table was preprocessed in two different ways in order to allow further processing using two different algorithms, the Bayesian Network algorithm and the Random Forest algorithm. The Bayesian Network algorithm does not allow analysis of numerical data. Therefore, numerical parameters were grouped based on clinical relevance as shown in Table 1. The grouping is done separately for each numerical parameter.

**Table 1:** Grouping of numerical data based on clinical relevance in order to allow analyzes using the Bayesian Network algorithm.

Body weight [kg]	0–7; > 7–15; > 15–30; > 30
Grouped age / grouped age at presentation / grouped age at first seizure [years]	≤ 7 kg: a (0–0.5); b (> 0.5–9), c (> 9–12); d (> 12)> 7–30 kg: a (0–0.5); b (> 0.5–6); c (> 6–10); d (> 10)> 30 kg: a (0–0.5); b (> 0.5–5); c (> 5–9); d (> 9)Boxer and French Bulldog: (0–0.5); b (> 0.5–4); c (> 4–6); d (> 6)
Number of seizures before first presentation	within the last 24 h: 0; 1; 2–3; 4–10; > 10with the last week: 0; 1; 2–3; 4–10; > 10within the last month: 0; 1; 2–3; 4–10; > 10within the last 6 months: 0; 1–4; 5–10; > 10within the last year: 0; 1–4; 5–10; > 10
Duration of seizure history before first presentation [months]	0–1; > 1–3; > 3–12; > 12

For the Random Forest model, one hot encoding was used for the features “first clinical signs observed by the owner” and “type of seizure” in order to adapt categorical data. The column “sex” was subdivided in two boolean columns “sex” and “castrated”.

### 2.3 Feature selection

After data preprocessing, a total of 33 features for the Random Forest algorithm and 17 for the Bayesian Network algorithm were available for analysis. In general, a smaller number of features is advantageous to achieve better results, since this reduces overfitting and generates more robust and more explainable models. Non-informative features can distract the model and may cause poorer results. Therefore, we focused on identifying features that are most relevant for the prediction. The following four feature selection methods were used: Permutation Importance, Forward Selection, Random Selection and Expert Opinion.

Permutation Importance measures the decrease in model score when a single feature value is randomly shuffled. During Forward Selection, a model is trained for every single feature. The feature that performed best and the corresponding accuracy score is stored. For the remaining features each is combined with the selected feature and a model is trained. The two features performing best are selected and the model score is stored. For the remaining features the process is continued until no feature is left. That way, the features performing best are added step wise. Finally, the scores of the model series are compared and the feature set of the model with the best score is chosen. In Random Selection, a subset of the features is randomly selected to train a model. The feature subset and the accuracy score are stored. The process is repeated 1,000 times and the feature subset with the best score is chosen. In Expert Opinion, the feature subset is selected by a clinician with 20 years of experience in this field.

For the Random Forest model, additional mean decrease in impurity (MDI), a feature importance score for tree models, was used for feature selection. MDI counts the times a feature is used to split a node, weighted by the number of samples it splits (10). Feature Selection based on MDI was utilized similar to Forward Selection, but the MDI score, previously calculated through training a model with all features, determined the order of the sequentially added features. For model selection, the accuracy score was used too.

### 2.4 Data analysis

For data analysis, we applied two machine learning algorithms, the Bayesian Network and the Random Forest. The Bayesian Network is a probabilistic graphical model that allows to compute probabilities between symptoms and diseases (11). Given clinical data (including symptoms), it allows to compute the probability of structural epilepsy. The Random Forest is a classifier which is based on multiple decision trees and is a widely used approach for this type of classification task (12).

We used the RandomForestClassifier from the package scikit-learn in Python with its default parameters (100 trees, Gini criterion to measure the quality of a split, no maximal tree depth, at least two samples for a split, at least one sample in a leaf, initialization with the same random state 0).

For model validation, we performed a 10-fold cross validation. A k-fold-cross validation is a resampling method for model validation. For k = 10, the data is split into 10 parts, whereas 9 parts of the data is used for training and one part for testing. This is repeated 10 times, resulting in 10 different collections of disjoint training and testing data sets. Thus, it is guaranteed that the trained models are never tested on the same data. The accuracy metric was used for model comparison and the best model was chosen.

## 3 Results

A total of 444 dogs of 119 different breeds were identified retrospectively between January 2017 and June 2021. Some dogs had to be removed because of incomplete data, whereas 279 dogs have been used for further analysis. An additional 49 dogs were included prospectively resulting in a total number of 328 dogs included. The most frequent breeds that were represented by at least 5 individuals were: mixed breed dog (*n* = 90), French bulldog (*n* = 39), Labrador retriever (*n* = 20), Chihuahua (*n* = 11), Bolonka zwetna (*n* = 9), Yorkshire terrier (*n* = 8), Beagle (*n* = 8), Pug dog (*n* = 7), Australian shepherd dog (*n* = 6), Jack Russell terrier (*n* = 6), Golden retriever (*n* = 5), and Great Swiss mountain dog (*n* = 5). The type and number of observed features in those 328 dogs are summarized in Table 2 for categorical features and Table 3 for numeric features. The statistics of the grouped features are in Table 1 of the supplementary.

**Table 2:** Features selected for training both models (Bayesian Network and Random Forest).

		All	Structural epilepsy	No structural epilepsy
n		328	102	226
Sex	Female intact	90	29	61
	Female spayed	45	18	27
	Male intact	135	38	97
	Male neutered	58	17	41
Neurological deficits on initial presentation		213	89	124
Lateralized neurological deficits on initial presentation		78	42	36
Cluster seizures		150	58	92
Status epilepticus		53	16	37
Clinical signs observed by the owner	Paroxysmal events	150	43	107
	Recumbency	47	16	31
	Abnormal behavior	38	14	24
	Salivation	24	13	11
	Gastrointestinal signs	15	4	11
	Vocalization	9	2	7
	Abnormal coordination	9	1	8
	Abnormal motor movements	6	2	4
	Head tremor	5	1	4
	Other clinical signs	25	6	19
Seizure types	Generalized seizures	279	86	193
	Focal seizures	27	6	21
	Generalized and focal seizures	22	10	12
Body weight	≤ 7 kg	70	22	48
	> 7 - ≤ 15 kg	88	33	55
	15 - ≤ 30 kg	107	26	81
	> 30 kg	63	21	42

**Table 3:** Comparison of numerical features between dogs with structural epilepsy and no structural epilepsy (*n* = 328) with median, range, and IQR (Q1–Q3).

	All dogs	Dogs with structural epilepsy	Dogs without structural epilepsy	*p*-value
Body weight [kg]	*m* = 15.55 [0.46–90] IQR: 8.3–27.5	*m* = 13.73 [1.77–49] IQR: 8.39–26.6	*m* = 16.55 [0.46–90] IQR: 8.21–27.6	0.6224
Age [days]	*m* = 3,656 [459–8,438] IQR: 2502–4,832	*m* = 4,781 [1275–7,969] IQR: 3781–5,692	*m* = 3,210 [459–8,438] IQR: 2317–4,170	1.65*10^−12^ (***)
Age at presentation [days]	*m* = 2008 [50–6,197] IQR: 939–3,287	*m* = 3,273 [79–5,866] IQR: 2309–4,253	*m* = 1,586 [50–6,197] IQR: 744–2,447	5.00*10^−16^ (***)
Age at first seizure [days]	*m* = 1,646 [48–6,197] IQR: 751–3,115	*m* = 3,214 [48–5,865] IQR: 2176–4,074	*m* = 1,214 [50–6,197] IQR: 521–2037	0.76*10^−18^ (***)
Seizures in last 24 h	*m* = 1 [0–60] IQR: 0–2	*m* = 1 [0–60] IQR: 0–3	*m* = 1 [0–12] IQR: 0–2	0.1468
Seizures in last week	*m* = 0 [0–60] IQR: 0–2	*m* = 1 [0–60] IQR: 0–2	*m* = 0 [0–12] IQR: 0–1	0.0003 (***)
Seizures in last month	*m* = 0 [0–60] IQR: 0–2	*m* = 0 [0–60] IQR: 0–1	*m* = 0 [0–30] IQR: 0–2	0.1402
Seizures in last 6 month	*m* = 0 [0–60] IQR: 0–2	*m* = 0 [0–60] IQR: 0–1	*m* = 1 [0–60] IQR: 0–2	0.0007 (***)
Seizures in last year	*m* = 0 [0–60] IQR: 0–2	*m* = 0 [0–60] IQR: 0–0	*m* = 0 [0–50] IQR: 0–1	0.0018 (**)
Duration of seizure history [days]	*m* = 29 [−271–3,249] IQR: 1–189.25	*m* = 7.5[−223–3,249] IQR: 1–52	*m* = 69 [−271–2,557] IQR: 1–309	0.0002 (***)

Comparison of medians between both groups was performed using the Mann–Whitney-U-Test. (*) *p* < 0.05, (**) *p* < 0.01, (***) *p* < 0.001.

**Figure 1:**
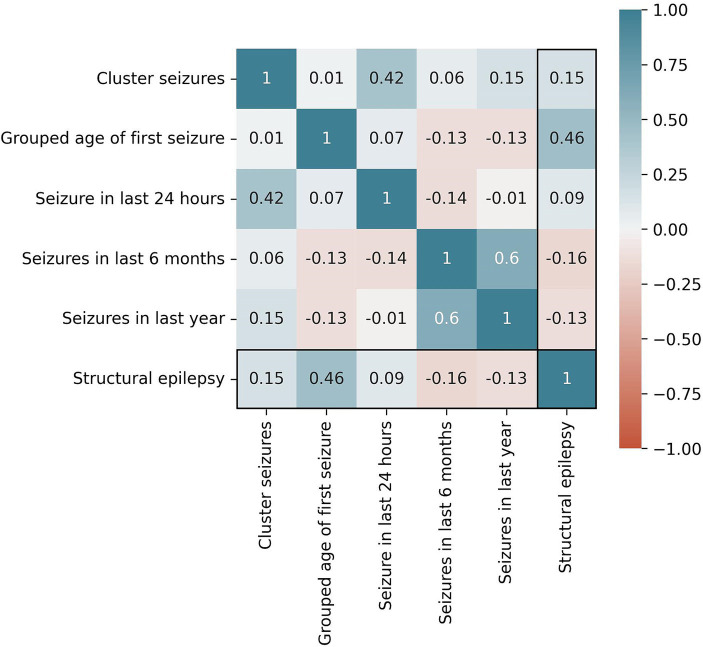
Correlation of selected features for the Bayesian Network algorithm and the target variable “structural epilepsy”. Cramer’s V is used as metric.

**Figure 2:**
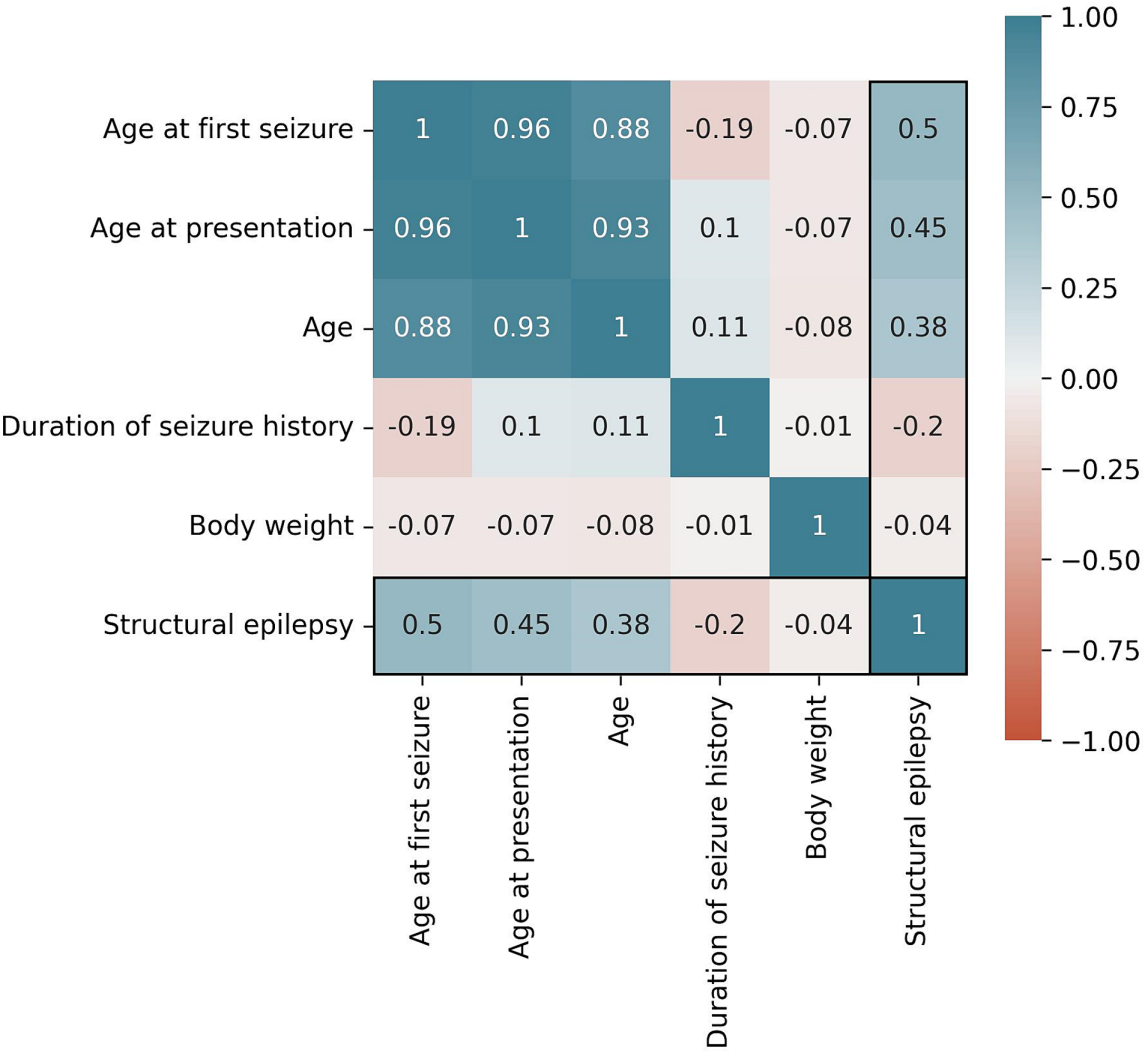
Correlation of selected features for the Random Forest algorithm and the target variable “structural epilepsy.” The Pearson correlation coefficient is used as metric.

In total, 89,848 models were trained. The following five features performed best for the Bayesian Network algorithm based on Random Selection: cluster seizures, grouped age of first seizure, seizure in last 24 h, seizure in last 6 months, and seizure in last year. The mutual correlations of these features as well as the target variable structural epilepsy are shown in Figure 1. For the Random Forest based on MDI feature selection, the following five features performed best: age at first seizure, age at presentation, age, duration of seizure history, and body weight. The mutual correlations of these features as well as the target variable structural epilepsy are shown in Figure 2.

The performance of the applied feature selection methods to predict structural epilepsy in dogs is shown in Table 4. The best results were obtained for the Bayesian Network combined with Random Selection as well as the Random Forest combined with MDI. Both approaches reached an accuracy of 0.969. The Bayesian Network reached slightly better values for sensitivity (0.857) and AUC (0.971). The selected features for all feature selection methods can be find in Table 2.

**Table 4:** Performance of the Bayesian Network and the Random Forest combined with the different feature selection methods to predict structural epilepsy (AUC: area under the curve; MDI: mean decrease in impurity).

		Permutation importance	Forward selection	MDI	Random selection	Expert opinion
Bayesian Network	Sensitivity	0.857	0.857	----	0.857	0.800
	Specificity	0.920	0.880	----	1.000	**1.000**
	Accuracy	0.906	0.875	----	0.969	0.938
	AUC	0.954	0.886	----	0.971	0.916
Random Forest	Sensitivity	0.714	0.714	0.800	0.714	**0.857**
	Specificity	0.960	1.000	1.000	0.960	0.960
	Accuracy	0.906	0.938	0.969	0.906	0.938
	AUC	0.923	0.909	0.937	0.957	0.911

**Figure 3:**
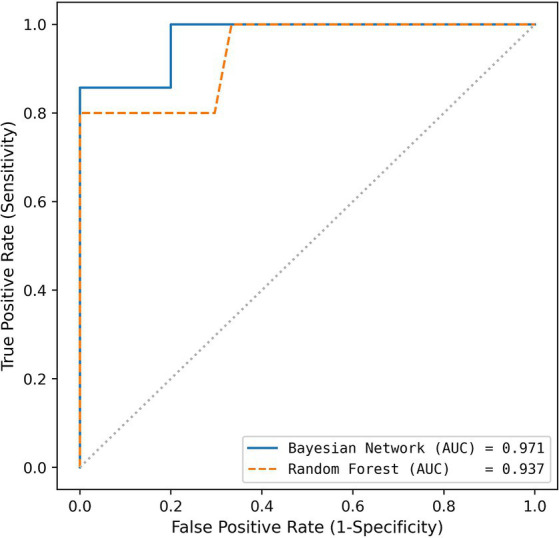
Receiver operating characteristics for both algorithms used (blue: Bayesian Network using Random Selection; orange: Random Forest using MDI Selection).

By varying the thresholds inside the two best performing models, the receiver operating characteristic (ROC) curves of the models can be obtained (Figure 3). The false positive rate (1 – specificity) on the x-axis is plotted against the true positive rate (sensitivity) on the y-axis. The ROC curves are both far away from the diagonal (dashed line), corresponding to a random classifier, and the Areas Under the Curve (AUC) are close to 1. By walking along the ROC curve (which is done by varying the threshold inside the model), a sensitivity of 1.000 can be obtained at the cost of a lower specificity (0.800 for Bayesian network and 0.667 for Random Forest).

**Figure 4:**
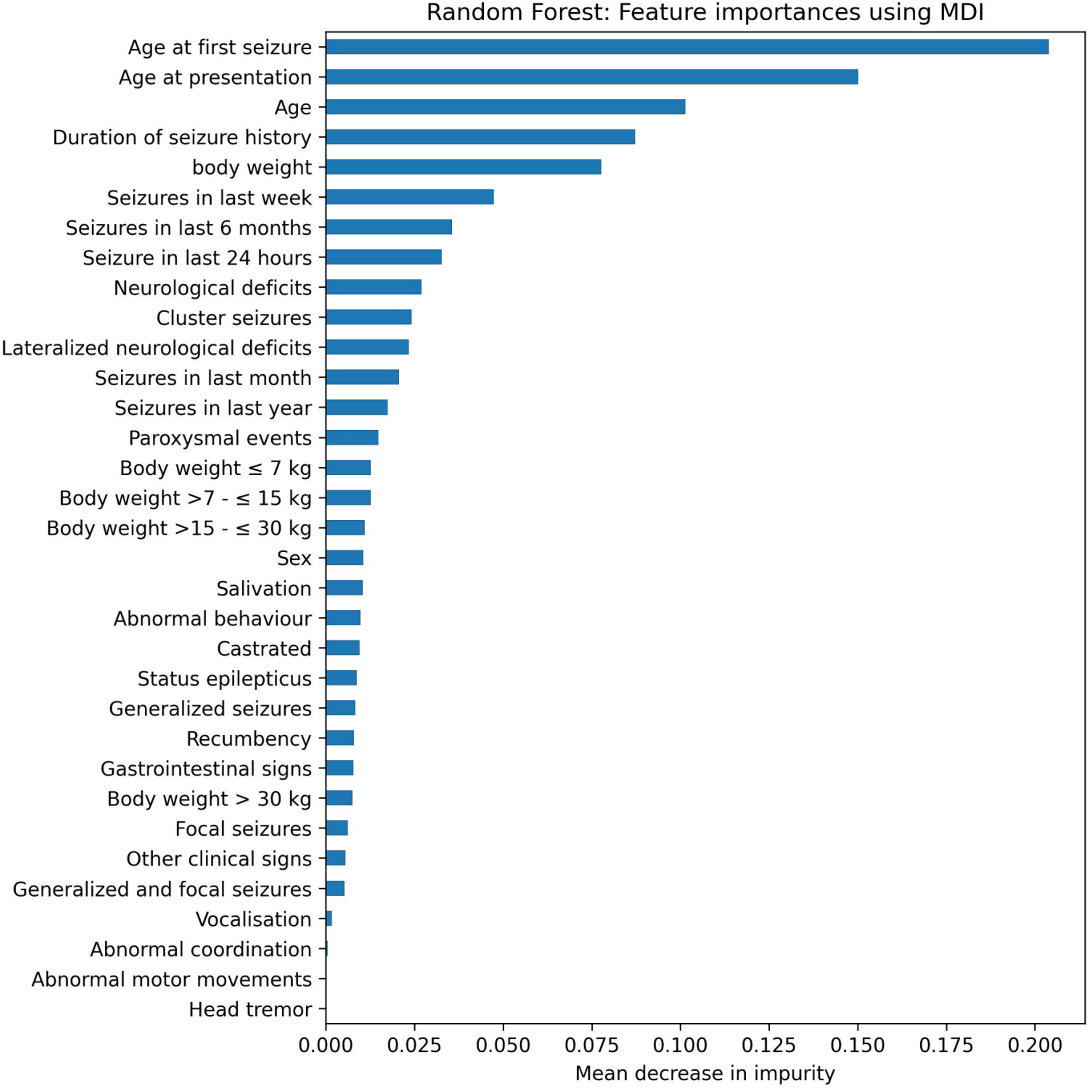
Feature importance using the Random Forest algorithms with Forward Selection in combination with the mean decrease in impurity (MDI).

The Random Forest computes a feature importance based on the Gini index for each available feature as a side result. These feature importance are shown in Figure 4 (for the Random Forest combined with the MDI score). The five most important features (MDI value >0.75) were selected by the feature selection algorithm. After that, the feature importance drops considerably. The performance of the model would deteriorate if more than the five most important features were used by the model.

## 4 Discussion

By applying the machine learning algorithms Bayesian Network, a sensitivity of 0.857 and a specificity of 1.000 resulting in a total accuracy of 0.969 could be obtained when classifying dogs with and without structural epilepsy. Thus, dogs with structural epilepsy and those without were identified correctly in about 97% of cases. The Random Forest model with MDI feature selection performed similar to the Bayesian Network. It reached also an accuracy of about 97% but with slightly smaller values for sensitivity and AUC than the Bayesian Network.

There are hardly any studies for comparison in veterinary medicine. However, these accuracies are higher than previously reported results, where ChatGPT did accurately identified structural epilepsy in 2 out of 5 dogs (1). These differences may be caused by the different approach applied in this study. ChatGPT that was used for prediction is a language processing model, that largely depends on random input made by internet users. It was applied to data without previous training. In contrast, in the study presented here, we have trained two different algorithms for prediction and the results were validated. In addition, the differences in accuracy to identify dogs with structural epilepsy may result from the large number of 238 dogs included here, whereas the study using ChatGPT looked at the small number (5 dogs) with structural epilepsy only (1).

Using artificial intelligence is an important step to support clinical reasoning. So far, most clinical decisions were based on clinicians’ expertise and personal experiences combined with knowledge derived from studies describing cohorts of patients. Attempts to predict the risk of certain intracranial pathologies in dogs with seizures were already made in the past. It was found that dogs having just one single seizure were less likely to suffer from a lateralized structural brain disease, whereas those dogs with abnormal findings on the neurological examination had a 16.5 times higher risk for such a lateralized lesion and a 12.5 times higher risk for a symmetrical structural lesion (13). However, that is a completely different approach than in our study, which was aiming for the prediction of the individual risk of dogs to suffer from structural epilepsy.

In the study presented here, two algorithms, the Bayesian Network and the Random Forest had been intentionally selected in order to make usage of the advantages of different algorithms. A Bayesian Network is a probabilistic graphical model. It treats uncertainty explicitly, and is thus suitable for small and incomplete data sets and inference with incomplete data. This algorithm provides a conditional probability distribution for every combination of variable values. However, the Bayesian Network requires discretization of continuous variables, hence it is poor in finding linear relationships, but it performs well if relationships between the variables are non-linear and complex.

The Random Forest model is an ensemble method using decision trees. It is robust to outliers and overfitting, can deal with linear and non-linear relationships and offers feature importance as a byproduct. It can handle numeric and categorical variables. It is not necessary to construct artificial categories when transforming numeric variables into categorical ones. To use categorical variables with equal order, these variables can be transformed with one hot encoding to boolean variables. Strongly correlated features are often problematic, however, Random Forest can handle them much better than linear regression models. If there are two strongly correlated features, the Random Forest algorithm randomly picks only one of them in a split. Thus, the correlated features are less likely to show up together at the same position in a tree.

For the reasons explained, we considered those two algorithms to be among the best for analyzing the data obtained in this study. However, it cannot be excluded that other algorithms could have resulted in an even higher diagnostic accuracy. We used the standard parameters when training the models. A hyperparameter tuning might be applied to further improve the classification results.

For any of the algorithms available, the decision on which features to be included is crucial (4). Feature selection allows to identify and rank the clinical parameters according to their importance with respect to the classification. Therefore, different features selection methods have been tested here, all of which have advantages and disadvantages. The Random Selection method can identify feature subsets consisting of low ranked single features but it does allow double selection. Forward Selection, however, provides good performing feature sets, where multiple solo ranked features may perform potentially better, but it can be a relatively slow model, whereas Permutation Importance is fast, easy to use and it is readily available since it is included in scikit-learn, a freeware online library for machine learning tools.

The Random Selection methods provided best results here if applied to the Bayesian Network or Random Forest algorithm. However, it cannot be excluded that other features that had been excluded during the initial phase of feature selection or features, that were not even collected could perform better. In addition, the number of dogs included, even though it appears relatively large for a veterinary study, was still not large enough to investigate certain features. Specifically, including the feature “breed” would have been very interesting, since clinical observation indicates that certain canine breeds are predisposed for specific structural intracranial diseases. For instance, Pug dogs and Yorkshire terriers are predisposed for necrotizing encephalitis, whereas Boxers and French bulldogs are more frequently affected by intracranial neoplasias (14–18). However, only 12 of the 88 different breeds were represented by at least 5 individuals and only 5 breeds were represented by at least 10 individuals. Therefore, a meaningful analysis of the feature “breed,” as interesting as it would have been, was not feasible.

Interestingly, the feature selection method Expert Opinion did not perform as good as the Random or MDI feature selection methods in identifying features to determine if dogs are affected by structural epilepsy, although an accuracy of about 94% was reached. It performed better than Forward Selection and Permutation Importance. This method is the closest of all methods to the current clinical decision-making process, where intracranial neoplasias are commonly associated with neurological deficits (specifically lateralized neurological signs) and therefore those had been included as features into the Expert Opinion model (13, 19). This discrepancy between clinical experience and a slightly lower accuracy of the Expert Opinion model may be at least partially explained by the fact that neoplasias in certain brain areas such as the olfactory and frontal lobes rarely cause interictal neurological deficits (18). In addition, pituitary neoplasia may be associated with rather unspecific signs such as disorientation or obtundation, that do not present any lateralization (20). Therefore, the importance of lateralized neurological deficits for prediction of structural epilepsy may have been overrated by the expert in the Expert Opinion feature selection method. That is supported by an only mid-range MDI of lateralized neurological deficits using the Random Forest algorithms with Forward Selection (Figure 4).

Of the 328 dogs, only 102 were diagnosed with structural epilepsy, therefore the target variable is slightly imbalanced. In order to also capture the performance of the model on the minority class, we used the metrics sensitivity, specificity, accuracy, did a ROC analysis, and computed AUC values. In some scenarios, a specificity of 1.000 was reached, but this often comes at the cost of lower sensitivity.

To avoid overfitting, feature selection and cross-validation were used. Feature selection acts as regularization, as only the most important features are selected for the final model. Cross-validation ensures that the trained model is always tested on test data that has not been used for training the model.

A weakness of this study is that some of the data was based on the observations made by the owners during collecting the clinical history of the pet. Different owners may have described the same clinical signs in a different way resulting in seemingly different observations. However, the data could only be extracted retrospectively from the patients’ files for most dogs based on the study designs. We have grouped some of this information during data processing in order to reduce the tremendous amount of different observations made by the owners for a meaningful analysis. Erroneous classification may have occurred in some cases during this grouping process that was done in order to transform individual verbal owner descriptions into repetitive clinical findings. That obstacle could be overcome in the future by providing pet owners and clinicians with a set of specific questions that have to be answered by selecting options from a predetermined menu. Another potential weakness of this study might be, that some data was based on the subjective assessment of the dogs’ neurological status by clinicians. Neurological examination performed by other clinicians may have resulted in slightly different findings. Therefore, the results presented here may not be safely applied to every other clinical setting. For further studies, it is desirable to identify features or feature expressions that can be repeatably and reliably obtained by different clinicians.

In conclusion, it can be said that structural epilepsy can be predicted with high sensitivity and specificity in dogs with seizures using machine learning algorithms. This information is not meant to replace further diagnostic tests in affected dogs, but it may rather facilitate client communication. The knowledge of the likelihood of structural epilepsy could be used as guidance to decide about appropriate diagnostic steps in dogs presented for seizures.

## Data availability statement

The raw data supporting the conclusions of this article will be made available by the authors, without undue reservation.

## Ethics statement

The animal studies were approved by Ethics Committee of the Veterinary Faculty of Leipzig University EK 2/2024. The studies were conducted in accordance with the local legislation and institutional requirements. Written informed consent was not obtained from the owners for the participation of their animals in this study because this study did retrospectively evaluate patient data available in the hospital data base.

## Author contributions

TF: Conceptualization, Formal analysis, Funding acquisition, Investigation, Methodology, Writing – original draft, Writing – review & editing. AN: Conceptualization, Data curation, Formal analysis, Methodology, Software, Validation, Writing – original draft, Writing – review & editing. AH: Investigation, Writing – original draft, Writing – review & editing. OK: Investigation, Validation, Writing – original draft, Writing – review & editing. SL: Investigation, Writing – original draft, Writing – review & editing. CT: Investigation, Writing – original draft, Writing – review & editing. SG: Investigation, Writing – original draft, Writing – review & editing. JD: Investigation, Writing – original draft, Writing – review & editing. LB: Investigation, Writing – original draft, Writing – review & editing. TK: Investigation, Writing – original draft, Writing – review & editing. VW: Investigation, Writing – original draft, Writing – review & editing. MK: Investigation, Writing – original draft, Writing – review & editing. IB: Investigation, Writing – original draft, Writing – review & editing. CM: Conceptualization, Formal analysis, Funding acquisition, Investigation, Methodology, Resources, Software, Supervision, Visualization, Writing – original draft, Writing – review & editing.

## References

[1] Abani S De Decker S Tipold A Nessler JN Volk HA. Can ChatGPT diagnose my collapsing dog? Front Vet Sci. (2023) 10:1245168. doi: 10.3389/fvets.2023.1245168, PMID: 37901112 PMC10600474

[2] Assi EB Nguyen DK Rihana S Sawan M. A functional-genetic scheme for seizure forecasting in canine epilepsy. (2018) IEEE trans. Biomed Eng. (2018) 65:1339–48. doi: 10.1109/TBME.2017.2752081, PMID: 28920893

[3] Barge P Oevermann A Maiolini A Durand A. Machine learning predicts histologic type and grade of canine gliomas based on MRI texture analysis. Vet Radiol Ultrasound. (2023) 64:724–32. doi: 10.1111/vru.13242, PMID: 37133981

[4] Brinkmann H Patterson EE Vite C Vasoli VM Crepeau D Stead M. Forecasting seizures using intracranial EEG measures and SVM in naturally occurring canine epilepsy. PLoS One. (2015) 10:e0133900. doi: 10.1371/journal.pone.0133900, PMID: 26241907 PMC4524640

[5] Feighelstein M Ehrlich Y Naftaly L Alpin M Nadir S Shimshoni I. Deep learning for video-based automated pain recognition in rabbits. Sci Rep. (2023) 13:14679. doi: 10.1038/s41598-023-41774-2, PMID: 37674052 PMC10482887

[6] Nejedly P Kremen V Sladky V Nasseri M Guragain H Klimes P. Deep-learning for seizure forecasting in canines with epilepsy. J Neural Eng. (2019) 16:036031. doi: 10.1088/1741-2552/ab172d, PMID: 30959492

[7] Spiteri M Knowler SP Rusbridge C Wells K. Using machine learning to understand neuromorphological change and image-based biomarker identification in cavalier king Charles spaniels with Chiari-like malformation-associated pain and syringomyelia. J Vet Intern Med. (2019) 33:2665–74. doi: 10.1111/jvim.15621, PMID: 31552689 PMC6872629

[8] Varatharajah Y Berry BM Worrell GA Brinkmann BH. Seizure forecasting and the Preictal state in canine epilepsy. Int J Neural Syst. (2017) 27:1650046. doi: 10.1142/S0129065716500465, PMID: 27464854 PMC5288735

[9] Berendt M Farquhar RG Mandigers PJJ Pakozdy A Bhatti SFM De Risio L. International veterinary epilepsy task force consensus report on epilepsy definition, classification and terminology in companion animals. BMC Vet Res. (2015) 11:182. doi: 10.1186/s12917-015-0461-2, PMID: 26316133 PMC4552272

[10] Lee C (2017) Feature importance measures for tree models part I. Available at: https://medium.com/the-artificial-impostor/feature-importance-measures-for-tree-models-part-i-47f187c1a2c3

[11] Niedermayer D. An introduction to Bayesian networks and their contemporary applications. In: Innovations in Bayesian networks: Theory and applications. eds. Holmes DE Jain LC Berlin: Springer (2008). 117–30.

[12] Ho TK. (1995). Random decision forests. Proceeding of 3rd International Conference on Document Analysis and Recognition 278–282.

[13] Armaşu M Packer RMA Cook S Solcan G Volk HA. An exploratory study using a statistical approach as a platform for clinical reasoning in canine epilepsy. Vet J. (2014) 202:292–6. doi: 10.1016/j.tvjl.2014.08.008, PMID: 25241948

[14] Elbert JA Yau W Rissi DR. Neuroinflammatory diseases of the central nervous system of dogs: a retrospective study of 207 cases (2008–2019). Can Vet J. (2022) 63:176–83. PMC8759338 35110776

[15] Higginbotham MJ Kent M Glass EN. Noninfectious inflammatory central nervous system diseases in dogs. Comp Contin Educ. (2007) 29:488–97. 17849703

[16] Higgins RJ Bollen AW Dickonson PJ Sisó-Llonch S. Tumors of the nervous system In: Meuten DJ, editor. Tumors in domestic animals. 5th ed. Ames, IA: Iowa State Press, Ames (2017). 834–91.

[17] Kishimoto TE Uchida K Chambers JK Kok MK Son NV Shiga T. A retrospective survey on canine intracranial tumors between 2007 and 2017. J Vet Med Sci. (2020) 82:77–83. doi: 10.1292/jvms.19-0486, PMID: 31801930 PMC6983661

[18] Miller AD Miller CR Rossmeisl JH. Canine primary intracranial Cancer: a Clinicopathologic and comparative review of glioma, meningioma, and choroid plexus tumors. Front Oncol. (2019) 9:1151. doi: 10.3389/fonc.2019.01151, PMID: 31788444 PMC6856054

[19] Snyder JM Shofer FS Van Winkle TJ Masicotte C. Canine intracranial primary neoplasia: 173 cases (1986–2003). J Vet Med Int Med. (2006) 20:669–75. doi: 10.1111/j.1939-1676.2006.tb02913.x, PMID: 16734106

[20] Menchetti M De Risio L Galli G Cherubini GB Corlazzoli D Baroni M. Neurological abnormalities in 97 dogs with detectable pituitary masses. Vet Q. (2019) 39:57–64. doi: 10.1080/01652176.2019.1622819 10.1080/01652176.2019.1622819 PMC6831018 31112462

